# Prostate‐specific antigen testing among young men: an opportunity to improve value

**DOI:** 10.1002/cam4.3800

**Published:** 2021-02-24

**Authors:** Suzanne M. Lange, Jacob P. Ambrose, Michael C. Flynn, William T. Lowrance, Heidi A. Hanson, Brock B. O’Neil

**Affiliations:** ^1^ Division of Urology University of Utah Salt Lake City UT USA; ^2^ Departments of Surgery and Population Sciences University of Utah Salt Lake City UT USA; ^3^ Department of Internal Medicine University of Utah Health Salt Lake City UT USA; ^4^ Division of Urology Huntsman Cancer Institute University of Utah Salt Lake City UT USA

**Keywords:** biomarkers, cancer education, clinical guidelines, prostate cancer, screening, urological oncology

## Abstract

**Introduction:**

Prostate cancer screening using prostate‐specific antigen (PSA) testing remains widespread. The prevalence of PSA testing in young men is unknown and may be an appropriate target for improving health care by decreasing low‐value testing in this age group. The purpose of this study was to determine PSA testing rates in men younger than current guidelines support.

**Materials and Methods:**

Health Informational National Trends Surveys (HINTS) from 2011 to 2014 and 2017 were analyzed to establish the prevalence of PSA testing in young men and to evaluate the differences in testing rates based on race.

**Results:**

The combined survey data included 5178 men, with 2393 reporting previous PSA screening. Of men ages 18–39, 7% recalled receipt of PSA testing. Twenty‐two percent of men between the ages of 40 and 44 had been tested. Among men under age 40, PSA testing was more common among black men (14%) compared to white men (7%), Hispanics (6%), and men of Asian descent (8%). Logistic regression modeling demonstrates that black men under the age of 40 were more likely to undergo PSA testing than other racial or ethnic groups (odds ratio 2.14; 95% CI 1.17, 3.93).

**Conclusions:**

Current guidelines do not recommend routine PSA testing in average‐risk men under the age of 40. This study found that a significant number of young men are exposed to testing, with the greatest risk among black men. This suggests that there is an opportunity to improve the value of PSA testing by decreasing testing in young men.

## Graphical abstract

Health Informational National Trends Surveys were analyzed to establish the prevalence of PSA testing in young men and to evaluate the differences in testing rates based on race. We found that a significant number of young men are exposed to testing, with the greatest risk among black men. These results suggest there is an opportunity to improve the value of PSA testing by decreasing testing in young men, and thereby decrease premature exposure to the emotional and financial burden of false positives and the physical risks of diagnostic testing and treatment.

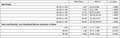

## 1 INTRODUCTION

Whether to pursue prostate‐specific antigen (PSA)‐based screening remains a topic of debate. However, there remains substantial agreement between guidelines that testing in certain men is low‐value care. Low‐value screening increases the risks for overtesting, overdiagnosis, and overtreatment. For example, screening in older men with short life expectancies is low‐value because many such patients may not experience the benefit of screening due to the indolent nature and long natural history of most prostate cancers. There have been multiple studies evaluating prevalence of PSA testing in older men, but none evaluate low‐value PSA practice patterns in younger men.^1^, ^2^, ^3^ The Center for Disease Control database identifies that those under the age of 45 have a low risk of prostate cancer. Between the ages of 40 and 49 the incidence is approximately 35 per 100,000 men, and for men below the age of 40, it is less than 1 per 100,000 men.^4^ In addition to testing in older men, testing for prostate cancer in young men may be another source of low‐value care that limits the overall benefits of PSA‐based prostate cancer screening. In this study, we sought to identify the prevalence of PSA testing in men younger than is recommended by current evidence‐based guidelines.

## 2 METHODS

### 2.1 Data

Data for this study were extracted from Health Information National Trends Surveys (HINTS) from 2011 to 2014 and 2017. HINTS is a cross‐sectional, nationally representative survey series developed through the National Cancer Institute.^5^ The data collected, via telephone or mailer questionnaire, surveys patients older than 18 regarding knowledge, perceptions, and use of cancer and health‐related information. We limited our analysis to male respondents under the age of 70, regardless of age, race, and health status. Respondent factors of interest included if they have ever had a PSA test, age, and race/ethnicity.

### 2.2 Analysis

Descriptive statistics of the respondent factors were stratified by whether respondents reported having a PSA test as well as by age group. The association of each factor with history of a PSA test (Y/N) was evaluated respectively for all encounters using multivariate logistic regression models adjusted for patient and provider factors. *P* values less than 0.05 were considered as statistically significant. All analyses were conducted in SAS (version 9.4).

## 3 RESULTS

The combined survey data included 5178 men, with 2393 reporting previous PSA screening. There were 1093 respondents between the ages of 18 and 39, with 78 (7.1%) reporting previous PSA testing (Table 1). Among men under 40, PSA testing was more common among black men (14%) compared to white men (7%), Hispanics (6%), and men of Asian descent (8%). Twenty‐two percent and 31% of men aged 40–44 and 45–49, respectively, reported previous PSA screening. The rate of PSA screening increased as age increased. The proportion of black men undergoing PSA screening exceeded that of all other races until the age group of 60–64, where the rate of screening in non‐Hispanic white men exceeded all others. Weighted estimates demonstrate that the number of men in the US under the age of 40 undergoing PSA screening is approximately 600,000 to 3.6 million.

**TABLE 1:** Descriptive statistics of PSA testing history stratified by age and race/ethnicity groups.

	Never been tested	Have been tested
Age group: <40		
Non‐Hisp White	558 (93.5%)	39 (6.5%)
Non‐Hisp Black or African American	96 (86.5%)	15 (13.5%)
Hispanic	213 (94.2%)	13 (5.8%)
Non‐Hisp Asian	79 (91.9%)	7 (8.1%)
Other	69 (94.5%)	4 (5.5%)
Total	1015 (92.7%)	78(7.3%)
Age group: 40–44		
Non‐Hisp White	196 (78.1%)	55 (21.9%)
Non‐Hisp Black or African American	38 (73.1%)	14 (26.9%)
Hispanic	66 (75.9%)	21 (24.1%)
Non‐Hisp Asian	24 (88.9%)	3 (11.1%)
Other	21 (77.8%)	6 (22.2%)
Total	348 (77.9%)	99 (22.1%)
Age group: 45–49		
Race/ethnicity		
Non‐Hisp White	205 (68.3%)	95 (31.7%)
Non‐Hisp Black or African American	41 (59.4%)	28 (40.6%)
Hispanic	67 (69.1%)	30 (30.9%)
Non‐Hisp Asian	30 (90.9%)	3 (9.1%)
Other	25 (73.5%)	9 (26.5%)
Total	368 (69.0%)	165 (31.0%)
Age group: 50–54		
Race/ethnicity		
Non‐Hisp White	206 (51.5%)	194 (48.5%)
Non‐Hisp Black or African American	40 (46.5%)	46 (53.5%)
Hispanic	55 (52.9%)	49 (47.1%)
Non‐Hisp Asian	13 (68.4%)	6 (31.6%)
Other	43 (55.8%)	34 (44.2%)
Total	357 (52.0%)	329 (48.0%)
Age group: 55–59		
Race/ethnicity		
Non‐Hisp White	163 (33.5%)	324 (66.5%)
Non‐Hisp Black or African American	30 (30%)	70 (70%)
Hispanic	44 (41.9%)	61 (58.1%)
Non‐Hisp Asian	17 (51.5%)	16 (48.5%)
Other	28 (40%)	42 (60%)
Total	282 (34.5%)	513 (64.5%)
Age group: 60–64		
Race/ethnicity		
Non‐Hisp White	135 (25%)	405 (75%)
Non‐Hisp Black or African American	33 (33.3%)	66 (66.7%)
Hispanic	34 (40.5%)	50 (59.5%)
Non‐Hisp Asian	10 (55.6%)	8 (44.4%)
Other	34 (38.2%)	55 (61.8%)
Total	246 (29.6%)	584 (70.4%)
Age group: 65–69		
Race/ethnicity		
Non‐Hisp White	92 (17.8%)	425 (82.2%)
Non‐Hisp Black or African American	25 (33.3%)	50 (66.7%)
Hispanic	22 (25.9%)	63 (74.1%)
Non‐Hisp Asian	9 (39.1%)	14 (60.9%)
Other	24 (24.7%)	73 (75.3%)
Total	172 (21.6%)	625 (78.4%)

Logistic regression results show that as a person moves up in age they are more likely to have had a PSA test (Table 2). For race/ethnicity in the group of men <40, 40–44, and 45–49, when compared to Caucasian men, analysis found no significant increase in risk for exposure to testing. When Black men are analyzed compared to all other races and ethnicities, those under 40 are more than twice as likely to undergo PSA testing. This increase in odds was not significant in ages 40–49.

**TABLE 2:** Logistic regression analysis of PSA testing by age and race/ethnicity.

	Odds ratio	95% CI	*p*‐value
Age group			
40–44 vs. <40	3.61	(2.62,4.98)	<0.0001
45–49 vs. <40	5.65	(4.2,7.59)	<0.0001
50–54 vs. <40	11.23	(8.51,14.81)	<0.0001
55–59 vs. <40	22.28	(16.93,29.32)	<0.0001
60–64 vs. <40	28.48	(21.58,37.6)	<0.0001
65–69 vs. <40	43.89	(32.85,58.63)	<0.0001
Race and ethnicity, non‐Hisp Black/African American vs. Other			
<40	2.141	(1.17, 3.93)	0.0141
40–44	1.38	(0.71, 2.68)	0.3421
45–49	1.669	(0.99, 2.82)	0.0553

## 4 DISCUSSION

This study demonstrates that numerous young men are exposed to PSA testing even prior to the youngest recommended age. Depending on the professional association, recommendations for initiating PSA testing for men at average risk range from 45 to 55; and, as young as 40 for those with high risk. High risk is typically defined as black men and those with strong family history of prostate cancer (i.e. early diagnosis, multiple family members, etc.).^6^, ^7^, ^8^ The indications for PSA testing in the young men of this study are unknown, but testing may be completed in those considered high risk, men with lower urinary tract symptoms, or those requesting testing. Additionally, testing in young men may be done as part of routine testing given the relative low cost, low risk, and ease of ordering in comparison to other cancer screenings (e.g. colonoscopy for colon cancer).

Regression models also demonstrated an increased risk of PSA testing as age increased, which is not surprising given the known increased prevalence of prostate cancer and the cumulative risk of PSA testing exposure as men age. However, black men under the age of 40 were most likely to undergo PSA testing suggesting a disparate exposure to guideline‐discordant testing among this group. Significant differences were not demonstrated with white men versus others despite age group; nor, in black men versus others in ages greater than 40.

There are limitations with this study. The nature of cross‐sectional analysis does not allow for an estimate of incidence of prostate cancer. Responses were self‐reported and were based on recollection of ever having been tested, so are subject to recall bias; however, this self‐reporting likely leads to lower estimates than the true rate of testing in young men. The indication for testing is also unknown. It may be that the testing in young men is done among those with genitourinary symptoms. Such testing in young men may reflect a misunderstanding of the role of PSA in the management of these symptoms in contrast to its use for the early detection of prostate cancer.^6^, ^9^ Another limitation of the study is the lack of clinical patient variables available in the HINTS database, as specific family history, patient diagnoses, and physician practice patterns would improve the understanding of why PSA testing is being used inappropriately. Further evaluation using a longitudinal commercial claims and encounter database is planned to validate the prevalence of testing in young men, expand the understanding of inappropriate PSA testing in this group, and characterize the downstream effects that may impact patients as a result of inappropriate testing.

A recent report demonstrated a rising incidence of prostate cancer in young men. Bleyer et al. found a 2% increase in prostate cancer incidence per year since 1990 in men ages 15–40. They also identified worse 5‐year survival and more aggressive disease when compared to men diagnosed at age 40 and older [8]. Despite this increase in prostate cancer among this age group, the overall incidence remains less than 1 case per million men and the most likely explanatory factor is increase in PSA screening rates. Despite a slight increase in early diagnoses, the overall low incidence rate suggests that PSA testing in this age group continues to be of low utility.

There is ongoing debate regarding the benefit of PSA screening, especially concerning decreases in prostate cancer specific mortality.^8^, ^10^, ^11^ Incidence of prostate cancer has correlated with the widespread use of PSA testing since the 1990s, and PSA testing appears to decrease the risk of metastatic disease.^4^, ^10^ Considering the ongoing debate of the benefits of prostate cancer screening, diagnosis, and treatment in groups of men with the highest incidence of prostate cancer, it is unlikely there is significant benefit from PSA testing in men under the age of 40. Even testing men under the age of 45 likely attributes little value and exposes these men to risks of physical, psychological, and financial harms. This analysis also demonstrated a disproportionate risk of PSA testing in young black men. While awareness of increased risk of prostate cancer in black men is important, this result suggests that young black men may be at a disproportionately higher risk of inappropriate testing.^12^ Taken together, these data represent an opportunity to improve the value of PSA testing through reducing unnecessary testing in young men.

## 5 DATA AVAILABILITY STATEMENT

The data that support the findings of this study are openly available at https://hints.cancer.gov/data/download‐data.aspx

## CONFLICT OF INTEREST

None.

## AUTHOR CONTRIBUTIONS

Suzanne M. Lange MD: formal analysis, investigation, methodology, project admin, writing—original draft, writing—review and editing. Jacob P. Ambrose MS: data curation, formal analysis, investigation, software, validation, visualization, writing—review and editing. Michael C. Flynn MD: conceptualization, supervision, writing—review and editing. William T. Lowrance MD MPH MBA: conceptualization, supervision, writing—review and editing. Heidi A. Hanson PhD: conceptualization, data curation, methodology, resources, supervision, visualization, writing—review and editing. Brock B. O’Neil MD: conceptualization, formal analysis, funding acquisition, investigation, methodology, project admin, resources, supervision, visualization, writing—original draft, writing—review and editing.

## ETHICAL APPROVAL

Analysis using the Health Information National Trends Survey meets criteria for non‐human subjects’ research by the University of Utah institutional review board. This analysis did not require review.
